# Complicated Littre's umbilical hernia with normal Meckel's diverticulum: A case report and review of the literature

**DOI:** 10.1016/j.ijscr.2021.106126

**Published:** 2021-06-18

**Authors:** Giuseppe Evola, Enrico Piazzese, Santo Bonanno, Carla Di Stefano, Giovanni Francesco Di Fede, Luigi Piazza

**Affiliations:** a General and Emergency Surgery Department, Garibaldi Hospital, Piazza Santa Maria di Gesù 5, 95100 Catania, Italy; b Department of Emergency Medicine, Garibaldi Hospital, Piazza Santa Maria di Gesù 5, 95100 Catania, Italy; c Department of Diagnostic Radiology, Santa Marta e Santa Venera Hospital, Via Caronia, 95024 Acireale, CT, Italy

**Keywords:** Meckel's diverticulum, Littre's hernia, Umbilical hernia, Emergency surgery, Case report

## Abstract

**Introduction and importance:**

A Littre's hernia (LH) is defined by the presence of Meckel's diverticulum (MD) in any kind of hernia sac. Preoperative diagnosis of LH is a challenge because of its rarity and the absence of specific radiological findings and clinical presentation. Surgery is the appropriate treatment of complicated LH that is an extremely rare condition with approximately 50 cases reported in the literature over the past 300 years.

**Case presentation:**

A 46-year-old Caucasian female was admitted to the Emergency Department with a two-day history of abdominal pain. Physical examination revealed an irreducible and painfull mass in umbilical region. Abdominal computed tomography scan showed the protrusion of greater omentum and small bowel loop through the umbilical ring. Laboratory tests were unremarkable. After diagnosis of strangulated umbilical hernia, the patient underwent exploratory laparotomy: the irreducible umbilical hernial sac was opened with presence of incarcerated and strangulated omentum and uncomplicated MD. Resection of incarcerated and ischemic greater omentum alone was performed. The postoperative course of patient was uneventful.

**Clinical discussion:**

Meckel's diverticulum (MD) is a vestigial remnant of the omphalomesenteric duct, representing the most common congenital malformation of the gastrointestinal tract. Preoperative diagnosis of LH is very difficult and surgery represents the correct treatment of complicated LH.

**Conclusion:**

LH represents an extremely rare complication of MD difficult to diagnose and suspect because of the lack of specific radiological findings and clinical presentation. Surgery represents the appropriate treatment of abdominal wall hernias and complicated MD.

## Highlights

• A Littre's hernia (LH) is defined by the presence of Meckel's diverticulum (MD) in any kind of hernia sac.
• LH is an extremely rare complication of MD and its preoperative diagnosis is very difficult.
• Surgery represents the appropriate treatment of abdominal wall hernias and complicated MD.
• Routine resection of uncomplicated MD is not advised and it should be based on identified MD’ s risk factors.

## 1 Introduction

A Littre's hernia (LH) is the protrusion of Meckel's diverticulum (MD) through a potential abdominal opening with a container sac [1,2]. MD is a vestigial remnant of the omphalomesenteric duct representing the most common congenital malformation of the gastrointestinal tract [3]. It is a true diverticulum and is located 7 to 200 cm proximal to the ileocecal valve, measuring 1 to 12 cm in length and 0.3 to 7 cm in diameter [4]. MD is usually asymptomatic being discovered incidentally during diagnostic or surgical procedures performed for other disorders like as acute appendicitis [4,5] [5,6], Crohn's disease or peptic ulcer disease. MD can present with multiple complications including lower gastrointestinal hemorrhage, intestinal obstruction [6,7], inflammation, perforation and malignant degeneration. The herniation of a MD was first described by the French Surgeon Alexis de Littré in 1700 [7] [8]. LH is an extremely rare condition found in only 1% of all cases of MD with approximately 50 cases reported in the literature over the past 300 years [8] [9]. Preoperative diagnosis of LH is a challenge because of its rarity and the absence of specific radiological findings and clinical presentation. Although surgery represents the correct treatment of complicated MD, a debate exists regarding the appropriate management of uncomplicated and incidentally discovered MD. A case of uncomplicated LH, incidentally discovered during emergency repair of a recurrent and strangulated umbilical hernia, is presented with review of the literature in accordance with SCARE 2020 criteria [9] [10]. The purpose of this case report is to remember that uncomplicated MD does not always require surgical treatment.

## 2 Presentation of case

**Fig. 1:**
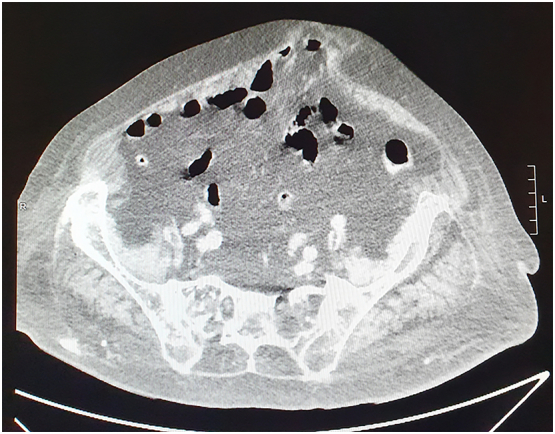
Preoperative abdominal computed tomography scan showing the protrusion of greater omentum and small bowel loop through the umbilical ring.

**Fig. 2:**
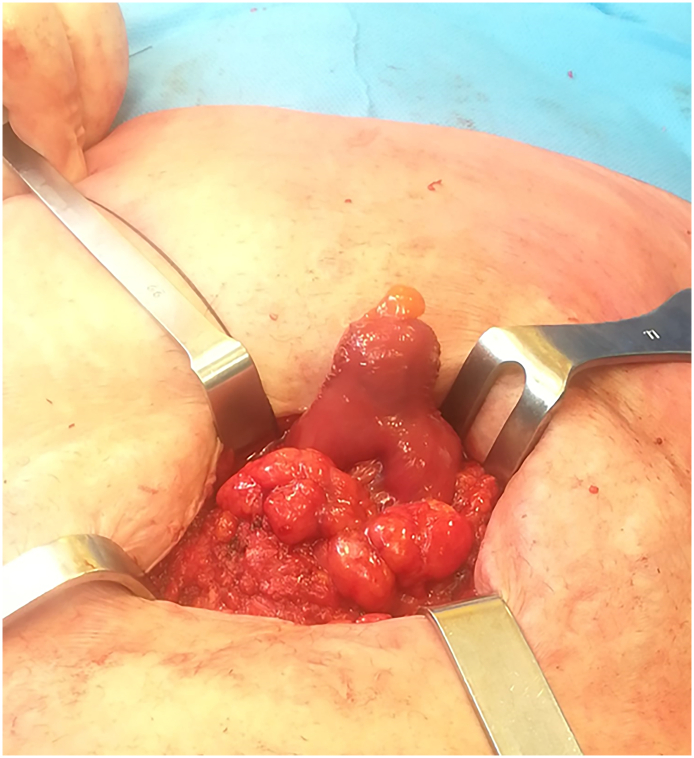
Irreducible umbilical hernial sac containing greater omentum and uncomplicated Meckel's diverticulum (LH): operative findings.

**Fig. 3:**
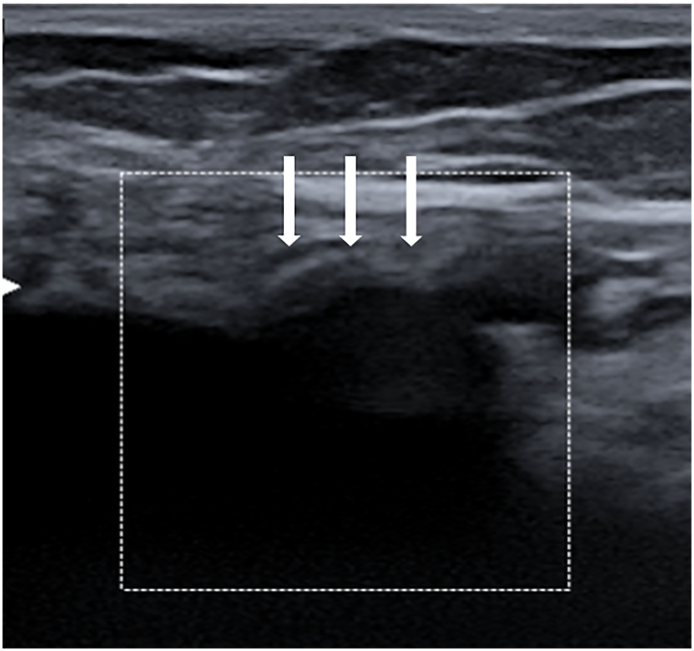
Postoperative abdominal wall sonography showing the correct position of the umbilical hernia mesh implant (white arrow).

A 46-year-old Caucasian female presented to the Emergency Department with a two-day history of abdominal pain. Vital signs on admission were normal. The patient had a history of previous umbilical hernia suture repair, her familial medical history was normal. She wasn't taking any drug, referred habit on smoking but denied alcohol consumption. She was employed by profession, married and of medium socio-economic status. On physical examination, an irreducible and painfull mass of 4 × 4 cm was observed in umbilical region, like as strangulated umbilical hernia, associated with generalized abdominal pain without Blumberg's sign. Abdominal computed tomography (CT) scan showed the protrusion of greater omentum and small bowel loop through the umbilical ring with a hernia gate of 3 cm (Fig. 1) without signs of intestinal obstruction. Laboratory studies were within normal limits. The patient, after understanding the severity of his medical condition and accepting surgery, was taken emergently to the operating room by experienced general surgeons (the first two authors) for surgical exploration of the umbilical hernia sac under general anesthesia. Prophylactic antibiotic (Ceftriaxone 1 g IV) was started 1 h before surgery. The patient was placed in the supine position on the operating table: intraoperatively the irreducible umbilical hernia sac was opened with presence of incarcerated and strangulated omentum and uncomplicated MD and measuring 2 by 2 cm (Fig. 2). Resection of incarcerated and ischemic greater omentum was performed and the small bowel loop bearing the uncomplicated MD was repositioned in the abdominal cavity. An intraperitoneal mesh (Composite Polyester Mesh with Absorbable Collagen Film, 12 cm round) was placed to cover the defect 4 cm beyond the edges of the umbilical hernia, attached with trans parietal points of absorbable suture to the abdominal wall. Patient was given received an IV injection of Ceftriaxone 1 g once daily for other four days. The postoperative recovery was uneventful and the patient was discharged on the 4th postoperative day in a stable condition. The patient tolerated the advice provided to avoid lifting for one month after surgery. Postoperative abdominal wall sonography was carried out at 3 months of the follow-up period showing the correct position of the mesh implant (Fig. 3). After a follow-up of 12 months the patient is asymptomatic.

## 3 Discussion

This report describes an extremely rare case of asymptomatic and uncomplicated MD incidentally discovered during emergency mesh repair of a recurrent and strangulated umbilical hernia. MD is the most common omphalomesenteric duct abnormality resulting from its incomplete obliteration. MD, found in approximately 2% of population, mainly occurs in children and rarely in adults [3]. It is a true diverticulum and is located 7 to 200 cm proximal to the ileocecal valve, measuring 1 to 12 cm in length and 0.3 to 7 cm in diameter [10]. Generally MD remains asymptomatic but sometimes it is manifested by its complications which occur occurring in 4–16% of cases [11]. Although prevalence of MD is equal between sexes, complications occur more often in males than females (M:F ratio 3:1) and decrease with age. The largest of the retrospective patient series (The Mayo Clinic Experience With 1476 Patients) identified four risk factors predisposing to symptomatic MD in adults: age younger than 50 years, male sex, diverticulum length greater than 2 cm, and the presence of histologically abnormal tissue [12]. The most common complication of MD in adults is intestinal obstruction, other complications are gastrointestinal bleeding, inflammation, perforation and malignant degeneration. LH represents an extremely a rare complication (1%) of MD that, located on because of its origin from the antimesenteric border of the ileum, is more prone to can protrude through any abdominal opening. Although symptomatic MD is more often seen in men, LH occurs more frequently in women (60.4%), mostly due to the high incidence of femoral and obturator Littre's hernias [8,9]. Approximately 50% of LH occurs in the inguinal region, 20% in the femoral region, 20% in the umbilical region and 10% in other locations [13]. The incidence of a LH presenting in complicated abdominal hernias has been reported to be 0.6% [14]. A MD can appear through a primary defect in the abdominal wall but also as a ventral hernia secondary to previous surgery. It may be accompanied in the sac by the ileal loop to which it is attached and rarely it may undergo incarceration, strangulation, necrosis or perforation [8,9]. In our case report an uncomplicated MD, surrounded by incarcerated and ischemic greater omentum, was seen intraoperatively within the recurrent and irreducible umbilical hernia sac. Diagnosis of LH is unlikely to be made preoperatively Preoperative diagnosis of LH is unlikely because of its low incidence and the absence of specific radiological findings and clinical. Despite the advances in radiological techniques, preoperative diagnosis of LH and its differentiation from other hernia are still impossible. Different imaging studies can be used for diagnosis of MD but the sensitivity and specificity is low [15]. In our case report abdominal CT scan showed a complicated umbilical hernia containing greater omentum and small bowel loop without identifying MD. Radiological exams generally show complications of MD leading to surgery and direct observation of complicated MD will yield the correct diagnosis. Symptoms and signs of LH are not specific and consist of vague crampy abdominal pain, dyspepsia, and occasional anorexia with malaise. In the case of incarcerated or strangulated LH the patient usually presents with an irreducible and painful mass and there may not be stigmata of bowel obstruction as only the MD but not the lumen of the bowel is involved in the hernia. Symptoms and signs of intestinal obstruction in LH are reported only in 34% of cases [16]. Common complications of LH are incarceration, strangulation and perforation. Clinical findings like incomplete manual reduction of an incarcerated hernia, fecal fistula in a hernia sac and previous history of rectal bleeding should alert the clinician about a LH [17]. The treatment of complicated LH is surgical and includes hernia defect repair and management of MD which depends on its clinical presentation [18]. The appropriate treatment of complicated MD is open or laparoscopic surgical resection including diverticulectomy, wedge resection or segmental bowel resection, depending on the type and integrity of diverticulum base and adjacent ileum as well the presence and location of ectopic tissue or tumor within MD. The presence and location of ectopic tissue cannot be accurately predicted intraoperatively by palpation or macroscopic appearance but can be predicted based on height-to-diameter ratio. Long diverticula (height-to-diameter ratio > 2) have ectopic tissue located at the body and tip; short diverticula have wide distribution of ectopic tissue including the base. If MD is long diverticulectomy should be performed, if MD is short or narrow-based, there is no palpable mass within, the same diverticulum may be managed by a simple wedge resection with a transverse closure of the remaining ileal defect. If the base of MD is broad, heterotopic tissue is palpated within MD or there are signs of inflammation, ischemia or perforation at the base of MD segmental small-bowel resection with anastomosis must be done. Malignant tumors require wide resection of the intestine and mesentery [19]. The presence of incarceration or perforation of MD and the possible filed contamination make difficult the use of mesh: in a systematic review of 53 cases, mesh was applied only in 17% of cases [16]. However a controversy exists about the correct management of an uncomplicated MD concerning its prophylactic resection when MD is incidentally discovered during surgery because of possible complications following its resection. Elective surgery is not recommended for cases where the diverticulum is discovered incidentally on radiological imaging. The treatment of complicated LH is surgical and includes removal of MD and hernia defect repair. MD should be resected with a transverse closure of ileum although a segmental small-bowel resection with anastomosis must be done if there are signs of inflammation, ischemia or perforation at the base of MD. The presence of incarceration or perforation of MD and the possible filed contamination make difficult the use of mesh: in a systematic review of 53 cases, mesh was applied only in 17% of cases [16]. Prophylactic resection of an incidentally discovered asymptomatic MD is debatable and is reasonable that the decision making of resection to be based on identified risk factors. In our case report we found an uncomplicated MD with a wide base and without a palpable mass suggesting ectopic tissue or tumor within the MD. We did not resect the uncomplicated MD, due to the presence of only one risk factor (age younger than 50 years) predisposing to symptomatic MD (17% of cases) and repair recurrent umbilical hernia with mesh. In our case report uncomplicated and incidentally discovered MD was not resected due to the presence of only one risk factor (age younger than 50 years) predisposing to symptomatic MD (17% of cases) and our decision to repair recurrent umbilical hernia with mesh.

## 4 Conclusion

LH represents an extremely rare complication of MD and remains mainly a subject of case report with approximately 50 cases reported in the literature over the past 300 years. Preoperative diagnosis of LH is difficult due to the absence of specific radiological findings and clinical presentation. Although surgery represents the appropriate treatment of abdominal wall hernias LH and with complicated MD, there is no consensus on the optimal treatment of uncomplicated MD: routine resection of MD is not advised and it should be based on identified risk factors because of possible complications following its resection.

## Sources of funding

This research did not receive any specific grant from funding agencies in the public, commercial, or not-for-profit sectors.

## Ethical approval

Ethical approval has been exempted by our institution because this is a case report and no new studies or new techniques were carried out.

## Consent

Written informed consent was obtained from the patient, for publication of this case report and accompanying images. A copy of the written consent is available for review by the Editor-in-Chief of this journal on request.

## Registration of research studies

Not applicable.

## Guarantor

Giuseppe Evola.

## Provenance and peer review

Not commissioned, externally peer-reviewed.

## CRediT authorship contribution statement

Giuseppe Evola: Operated on the patient, drafting the manuscript, literature research.

Enrico Piazzese: Operated on the patient, drafting the manuscript.

Santo Bonanno: Drafting the manuscript, literature research.

Carla Di Stefano: Drafting the manuscript and literature research.

Giovanni Francesco Di Fede: Drafting the manuscript and literature research.

Luigi Piazza: Revising the manuscript.

## Declaration of competing interest

The authors have no conflict of interest to declare.
